# Prevalence of Multiple Chronic Conditions Among Older Adults in Florida and the United States: Comparative Analysis of the OneFlorida Data Trust and National Inpatient Sample

**DOI:** 10.2196/jmir.8961

**Published:** 2018-04-12

**Authors:** Zhe He, Jiang Bian, Henry J Carretta, Jiwon Lee, William R Hogan, Elizabeth Shenkman, Neil Charness

**Affiliations:** ^1^ School of Information Florida State University Tallahassee, FL United States; ^2^ Department of Health Outcomes and Biomedical Informatics University of Florida Gainesville, FL United States; ^3^ Department of Behavioral Sciences and Social Medicine Florida State University Tallahassee, FL United States; ^4^ Department of Statistics Florida State University Tallahassee, FL United States; ^5^ Department of Psychology Florida State University Tallahassee, FL United States

**Keywords:** medical informatics, chronic disease, comorbidity, geriatrics

## Abstract

**Background:**

Older patients with multiple chronic conditions are often faced with increased health care needs and subsequent higher medical costs, posing significant financial burden to patients, their caregivers, and the health care system. The increasing adoption of electronic health record systems and the proliferation of clinical data offer new opportunities for prevalence studies and for population health assessment. The last few years have witnessed an increasing number of clinical research networks focused on building large collections of clinical data from electronic health records and claims to make it easier and less costly to conduct clinical research.

**Objective:**

The aim of this study was to compare the prevalence of common chronic conditions and multiple chronic conditions in older adults between Florida and the United States using data from the OneFlorida Clinical Research Consortium and the Healthcare Cost and Utilization Project (HCUP) National Inpatient Sample (NIS).

**Methods:**

We first analyzed the basic demographic characteristics of the older adults in 3 datasets—the 2013 OneFlorida data, the 2013 HCUP NIS data, and the combined 2012 to 2016 OneFlorida data. Then we analyzed the prevalence of each of the 25 chronic conditions in each of the 3 datasets. We stratified the analysis of older adults with hypertension, the most prevalent condition. Additionally, we examined trends (ie, overall trends and then by age, race, and gender) in the prevalence of discharge records representing multiple chronic conditions over time for the OneFlorida (2012-2016) and HCUP NIS cohorts (2003-2013).

**Results:**

The rankings of the top 10 prevalent conditions are the same across the OneFlorida and HCUP NIS datasets. The most prevalent multiple chronic conditions of 2 conditions among the 3 datasets were—hyperlipidemia and hypertension; hypertension and ischemic heart disease; diabetes and hypertension; chronic kidney disease and hypertension; anemia and hypertension; and hyperlipidemia and ischemic heart disease. We observed increasing trends in multiple chronic conditions in both data sources.

**Conclusions:**

The results showed that chronic conditions and multiple chronic conditions are prevalent in older adults across Florida and the United States. Even though slight differences were observed, the similar estimates of prevalence of chronic conditions and multiple chronic conditions across OneFlorida and HCUP NIS suggested that clinical research data networks such as OneFlorida, built from heterogeneous data sources, can provide rich data resources for conducting large-scale secondary data analyses.

## Introduction

### Background

Chronic conditions (CCs) affect nearly half of the adult population in the United States. The prevalence of some CCs such as hypertension, asthma, cancer, and diabetes has increased over the last a few years [1-3]. Older patients with multiple chronic conditions (MCCs) are often faced with increased health care needs and subsequent higher medical costs, posing significant financial burden to patients, their caregivers, and the health care system.

Understanding the trends in the prevalence of MCC informs policy makers, health care providers, and payers about chronic disease management and prevention and helps to predict future health care needs [4]. The literature on MCC research mostly uses national claims data or national surveys to estimate the prevalence of MCCs [4-7]. Freid et al [4] presented the estimates of the population aged 45 and older with 2 or more self-reported CCs using the National Health Interview Survey (NHIS) data. They reported that the percentage of adults with MCCs increased in both 45 to 64 years and 65 and older age groups between 1999 and 2010. Ward and Schiller [5] analyzed the prevalence of MCCs among US adults also using the 2010 NHIS data and reported an increasing prevalence of MCCs from 2001 to 2010. Ashman and Beresovsky did an MCC analysis among US adults who visited physician offices, using the National Ambulatory Medical Care Survey data [6]. They found that hypertension was the most prevalent CC that appeared in the top 5 MCC dyads and triads. He et al [7] used the National Health and Nutrition Examination Survey data and a public clinical trial registry—ClinicalTrials.gov—to analyze the gap between the prevalence of MCCs and the clinical trials on the prevalent MCCs. They found that the current and past clinical trials rarely investigate the prevalent MCCs.

Recent years have witnessed a wide adoption of electronic health record (EHR) systems driven by the Health Information Technology for Economic and Clinical Health (HITECH) Act of 2009 [8]. By 2015, over 90% of nonfederal acute care hospitals adopted a certified EHR [9]. By the end of 2017, about 90% of the office-based physicians have been using EHRs in the Unites States [10]. With public health reporting as part of the meaningful use criteria for hospitals to receive the incentive payments of the HITECH Act, EHRs have been recognized as an important data source for public health surveillance [11] (especially in chronic disease surveillance [12-14]), cohort identification for clinical studies [15], and disease-risk prediction [16]. The advantage of using EHRs over survey data is multifaceted. First, EHRs have fine-grained clinical data that are rarely collected and reported in the survey or claims data. Second, EHRs contain longitudinal patient data, whereas survey data mostly provide merely a snapshot of the health conditions for a person. However, as EHR data only contain patients who paid a visit to the health care facilities, they may not be as representative of the national population as the survey data. Therefore, it is necessary to investigate the extent to which EHR data can represent the broader population to inform researchers who are using EHRs for public health and chronic disease surveillance. Recently, Perlman et al created an EHR-based public health surveillance system in New York City [14]. They compared the CC estimates generated in this system with those from a population-based survey in New York and found that diabetes, hypertension, smoking, and obesity prevalence was close to the survey results, but depression and influenza vaccination estimates were substantially lower than the survey-based estimates [14].

The last few years have witnessed an increasing number of clinical research networks focused on building large collections of clinical datasets from EHRs and claims to offer a collaborative environment for researchers across disparate organizations. It is anticipated that the analysis of such data will lead to advances in medical knowledge, progress in health care delivery, and improvements in population health [17-21]. One notable example is the National Patient-Centered Clinical Research Network (PCORnet) [17,22], funded by the Patient-Centered Outcomes Research Institute (PCORI). PCORnet comprises a coordinating center and 33 partner networks, including 13 Clinical Data Research Networks (CDRNs) and 20 Patient-Powered Research Networks. PCORnet is “designed to make it faster, easier, and less costly to conduct clinical research than is now possible by harnessing the power of large amounts of health data and patient partnerships” [22]. It is a national “network of networks” that routinely collects data from a variety of health care organizations, including hospitals, community clinics, health plans, and national data registries (eg, cancer registries and vital statistics).

PCORnet empowers individuals and organizations to use this big dataset to answer practical questions that help patients, clinicians, and other stakeholders make informed health care decisions. For example, PCORnet provides an invaluable cohort discovery service that proves particularly useful for identifying cohorts of a variety of health conditions, especially for rare diseases. With such a large collection of electronic patient data, PCORnet can effectively support large-scale randomized clinical trials, comparative effectiveness research studies, and longitudinal observational studies. EHRs such as those warehoused in CDRNs have been widely used for comparative effectiveness analysis [23-26], cohort identification [27-29], and public health surveillance studies [25,30,31]. However, it is not yet known the extent to which the population in these CDRNs such as OneFloridais is representative of the national population. This is an important metric that needs to be examined to understand the comprehensiveness of the OneFlorida population now and to improve the interpretability and generalizability of the OneFlorida data and the reproducibility of the aforementioned studies.

Florida has the largest elderly population in the United States. OneFlorida is one of the 13 CDRNs contributing to the national PCORnet [32]. The OneFlorida Data Trust is a secure centralized data repository that integrates various data sources from contributing organizations in the OneFlorida research consortium, including 22 hospitals and 914 community-based clinical practices that provide care to 48% of Floridians. As of June 2017, the Data Trust contains 10.9 million patient records including data from partners’ EHR systems, as well as claims data from Florida Medicaid. Ultimately, the Data Trust will include claims data for Florida Medicare beneficiaries, Florida Vital Statistics records, and Florida Cancer Data System records. The OneFlorida Data Trust employs the PCORNet Common Data Model (CDM) version 3.1 [33], which uses standard vocabularies to encode diagnoses (ie, International Classification of Diseases, ICD), procedures (ie, ICD procedure codes, Current Procedural Terminology, and Healthcare Common Procedure Coding System codes), laboratory observations (ie, Logical Observation Identifiers Names and Codes), and medications (ie, RxNorm and National Drug Code). The OneFlorida and PCORnet data only contains Health Insurance Portability and Accountability Act limited data, for which we obtained permission to use. Throughout this paper, OneFlorida refers to the inpatient data extracts used to conduct this analysis unless otherwise noted.

### Objective

The purpose of this study is to estimate and compare the prevalence of common CCs and MCCs among older adults in Florida and United States from the OneFlorida Data Trust and a national data source—the National Inpatient Sample (NIS) from the Healthcare Cost and Utilization Project (HCUP) of Agency for Healthcare Research and Quality [34]. The NIS is a comprehensive source of inpatient hospital data in the United States. As NIS contains only the inpatient data, we also used the inpatient EHR records in the OneFlorida Data Trust to estimate Florida population. For this paper we define MCC as 2 or more CCs according to the Center for Medicare and Medicaid Services (CMS) algorithm [35].

We formulated 2 research questions (RQs) in this study:

RQ1: What is the prevalence of common CCs in hospital discharge records for older adults in the OneFlorida Data Trust inpatient data and how does it compare with unweighted national estimates from the HCUP NIS?

RQ2: Are the 10 most common CCs and the prevalence of MCC in hospital discharge records for older adults in the OneFlorida Data Trust consistent with the unweighted HCUP NIS national population?

## Methods

### Data Collection and Preparation

OneFlorida inpatient discharge records for 2012 to 2016 for 22 CCs were identified using the CMS Chronic Condition Warehouse (CCW) algorithm [35]. We included records with an admission source of home, another facility, or the emergency department. The 2013 discharge records were used for the cross-sectional analysis and the 2012 to 2016 records were used for a longitudinal comparison.

NIS is the largest publicly available all-payer inpatient health care database in the United States. Unweighted, it contains 7 million hospital discharge records each year and the weighted sample represents 25 million discharges. Beginning within the 2012 data year, the NIS approximates a 20% stratified sample of all discharges from US community hospitals, excluding rehabilitation and long-term acute-care hospitals. The 2013 NIS file was used for our cross-sectional analysis, and the 2003 to 2013 data were used for the longitudinal analysis. NIS includes information on all patients, including individuals covered by Medicare, Medicaid, or private insurance, uninsured. Researchers and policy makers use NIS to make national estimates of inpatient health care utilization [36], access to care [37], inpatient charges [36,38,39], quality of hospital care [37], and outcomes [39,40].

**Figure 1:**
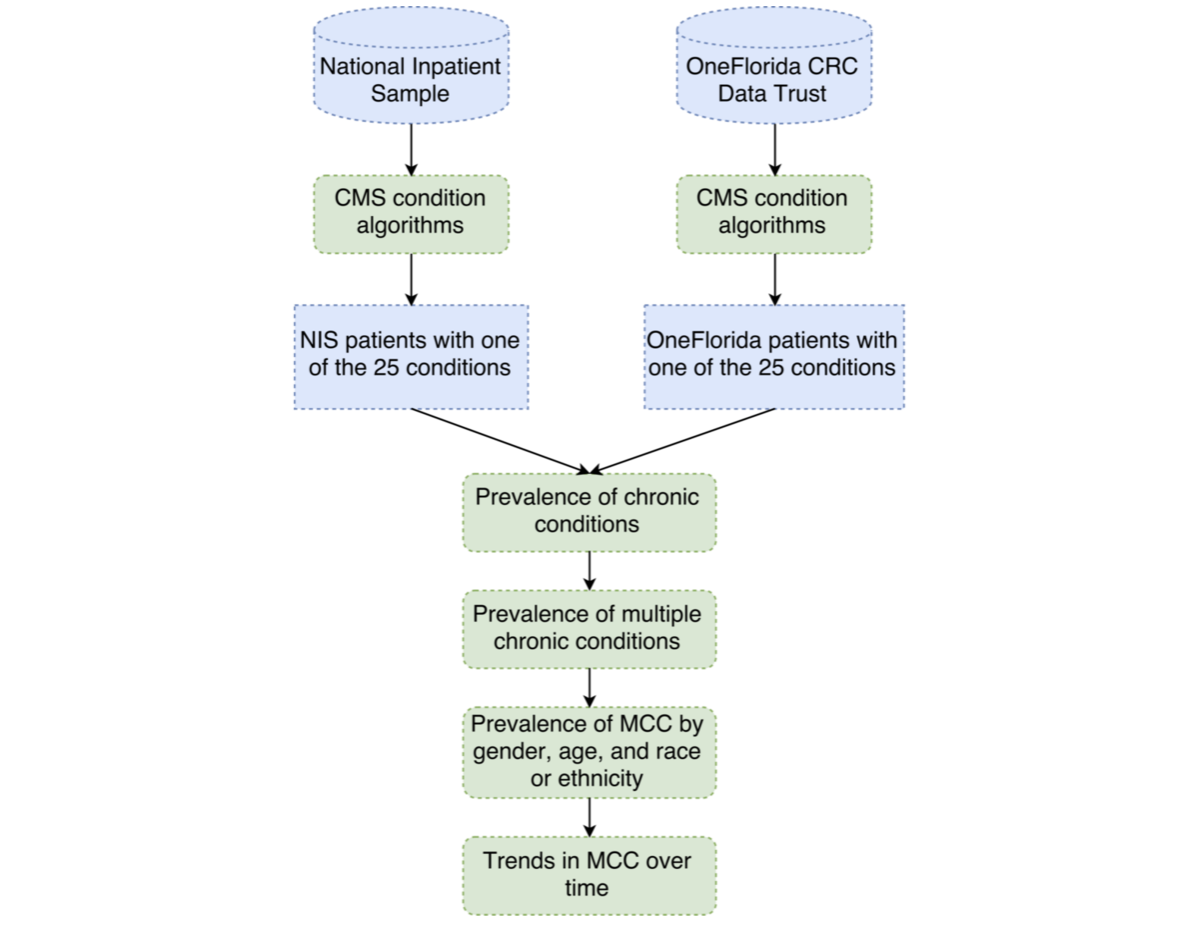
Workflow of data preparation and analysis. CRC: Clinical Research Consortium. CMS: Center for Medicare and Medicaid Services. NIS: National Inpatient Sample. MCC: multiple chronic conditions.

Figure 1 illustrates the process of data preparation and analysis. As the first step, we identified patients with CC using the CMS CCW algorithm [35]. The CMS CCW algorithm identifies cases for 27 condition categories using the criteria, such as (1) a validated list of ICD-9-CM and ICD-10 diagnosis codes, (2) the number of discharge record occurrences with diagnosis codes meeting the case definition within a year, (3) the number of consecutive years with confirming diagnoses in order to identify an individual case within a specific CC category in a given year to identify 27 conditions, and (4) the source type of service. We excluded 2 algorithm conditions that do not use inpatient records for case identification for cataracts or glaucoma, because those conditions are typically not associated with inpatient care. We modified the algorithm criteria for 7 other conditions, which were (1) rheumatoid arthritis and osteoarthritis, (2) chronic kidney disease, (3) heart failure, (4) diabetes, (5) Alzheimer disease, (6) Alzheimer disease and related conditions, and (7) ischemic heart disease. These 7 conditions require 2 or 3 consecutive years with the diagnosis to meet the case criteria or in the case of rheumatoid arthritis or osteoarthritis, 2 diagnoses within a year. Due to privacy concerns, the NIS does not assign unique patient identifiers that can be tracked across facilities or time. Therefore, we modified the criteria for those 7 conditions and identified cases based on a single inpatient discharge record. We limited the analysis to persons aged 65 years or older for the 25 remaining conditions defined by the CMS algorithm [35].

We identified older adults as those who were above 65 years at the time of inpatient discharge in both data sources. We stratified our analysis by age group, namely, 65 to 74, 75 to 84, and 85 and above. Besides age, we also extracted the gender and race or ethnicity variables of the patients. For OneFlorida analysis, we generated 2 datasets, one for a cross-sectional analysis (2013) and the other for a longitudinal analysis with data from all the years currently available in the OneFlorida Data Trust (2012-2016). For HCUP NIS, we used the 2013 data for the cross-sectional analysis and 2003 to 2013 data for the longitudinal analysis. The decision of using different year range for OneFlorida Data Trust and HCUP NIS was made based on the availability of the data and the richness of the analysis.

Nevertheless, both OneFlorida Data Trust and HCUP NIS have the 2012 to 2013 data.

OneFlorida Data Trust uses the PCORNet CDM version 3.1, which is a relational schema. The data are stored in a Microsoft SQL server hosted by the University of Florida Health Science Center. We included patients who had either direct inpatient admissions or emergency-to-inpatient admissions. The HCUP NIS data were released in the SAS format. We preprocessed the HCUP SAS datasets and loaded them into a Microsoft SQL server.

### Data Analysis

The analysis included descriptive statistics for the 25 individual conditions and MCC in 3 analytic files, that is (1) the OneFlorida data for the year 2013 only (OneFlorida 2013), (2) the NIS data for the year 2013 only (NIS 2013), and (3) the OneFlorida data for 2012 to 2016 (OneFlorida 2012-2016). Chi-square tests were used to examine group differences in the proportions of interest across the 3 data files.

We first analyzed the basic demographic characteristics of the older adults in the two 2013 datasets and the OneFlorida 2012 to 2016 data. Then we analyzed the prevalence of each of the 25 CCs for each of the 3 datasets. We did a deep dive, stratified the analysis, of the older adults with hypertension, the most prevalent condition. The prevalence of hypertension in the 24 age-gender-race-ethnicity strata was compared across the 3 datasets. We also examined the number of conditions per hospital record in each dataset for 2013 and further stratified the prevalence of patients with MCCs in 2013 by gender and race or ethnicity.

Additionally, we examined trends (ie, overall trends and then by age, race, and gender) in the prevalence of discharge records representing MCCs across time for the OneFlorida and NIS cohorts. Pearson correlation coefficient was computed to compare the MCC trends stratified by age group, sex, and race or ethnicity.

## Results

### Basic Characteristics

Table 1 shows the basic characteristics of the older adult populations in the OneFlorida 2013 data, OneFlorida 2012 to 2016 data, and the HCUP NIS 2013 datasets. The average age of older adults in the OneFlorida 2013 and 2012 to 2016 data was similar (2-tailed *t* test, degrees of freedom=63435.3226, *P*>.05). The older adults in OneFlorida 2013 were slightly younger than those in NIS 2013. There were more elderly female patients than elderly male patients across all 3 datasets. OneFlorida 2012 to 2016 had a statistically significantly higher percentage of Hispanics, non-Hispanic (NH) blacks, and a lower percentage of non-Hispanic whites and Asian or Pacific Islanders than the NIS 2013 (chi-square statistics 72587091891.83, *P*<.001).

### Prevalence of Chronic Conditions

The rankings of the top 10 prevalent conditions were the same across the 3 datasets. These conditions were hypertension, hyperlipidemia, ischemic heart disease, diabetes, anemia, chronic kidney disease, atrial fibrillation, heart failure, chronic obstructive pulmonary disease, and RA. However, there were differences in the prevalence of each disease between the NIS and OneFlorida data. Comparing the NIS and OneFlorida data, one can observe that a higher percentage of older adults in OneFlorida had hypertension (80.97% vs 76.32%), hyperlipidemia (52.42% vs 45.94%), and diabetes (35.32% vs 33.93%) than in NIS; whereas a higher percentage of older adults in NIS had chronic kidney disease (33.22% vs 31.24%) and heart failure (25.36% vs 19.77%). The prevalence of arthritis was 43% in male and 54% in female respondents in a recent national survey of older adults (65 and older) with self-reported chronic medical conditions in 2013 to 2014 [3]. The numbers were nearly twice the prevalence of such a condition in the inpatient clinical data reported in Table 2. This likely reflects the fact that people with arthritis were mostly treated in outpatient settings and thus diagnosis of arthritis is irrelevant to most inpatient discharges.

### Prevalence of Hypertension by Gender, Age Group, and Race or Ethnicity

Table 3 shows the prevalence of hypertension in older adults stratified by sex, age group, race and ethnicity in the NIS 2013, the OneFlorida 2013, and the pooled OneFlorida 2012 to 2016 data. Hypertension was chosen because it was the condition with the highest prevalence among the older persons we studied. The largest differences in the estimates between the 2013 files (OneFlorida and NIS) was about 3% for NH white females aged 85 years and older, and NH white males aged 65 to 74 years. We observed differences of more than 1% for females in the following 4 strata—NH black aged 65 to 74 years, NH white aged 65 to 74 years, NH white aged 75 to 84 years, and NH white aged 85 years and older. Among males, differences of greater than 1% were observed for the strata except for NH white aged 75 to 84 years. Estimates between OneFlorida 2013 and the pooled OneFlorida 2012 to 2016 data were largely similar with some increases in OneFlorida 2012 to 2016 data for hypertension prevalence, perhaps reflecting the increasing trends associated with obesity and sedentary life styles.

### Prevalence of Multiple Chronic Conditions

**Figure 2:**
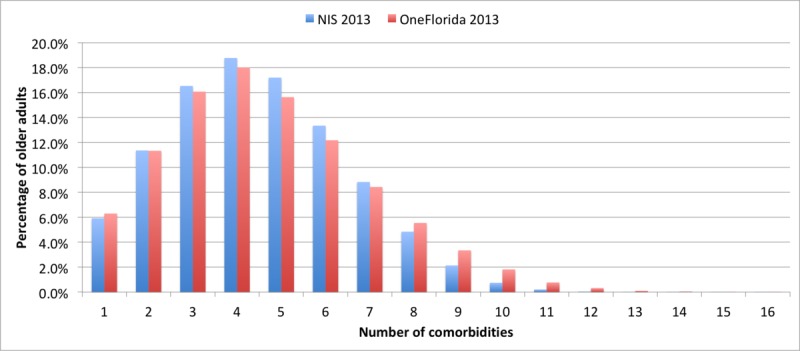
Number of conditions in older adults in the Healthcare Cost and Utilization Project National Inpatient Sample (NIS) 2013 and OneFlorida2013 data.

Figure 2 illustrates the percentage of the population with one or more CCs, which is, MCCs in older adults in the HCUP NIS and OneFlorida for 2013. The 3 datasets exhibited similar characteristics. Out of the 25 CCs, more than 18% older adults had 4 conditions. More than 65% older adults had 4 or more conditions. Persons with MMCs were very prevalent among older Americans.

### Prevalence of Multiple Chronic Conditions by Gender

**Figure 3:**
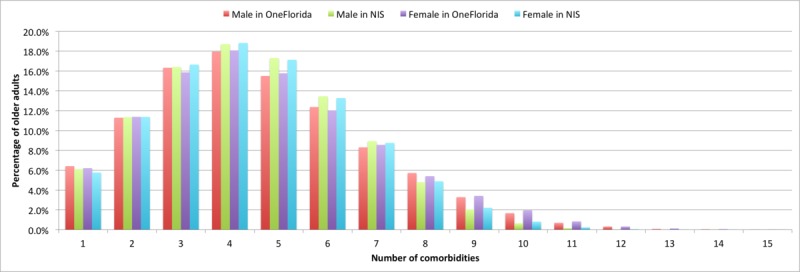
Prevalence of multiple chronic conditions in older adults by gender in Healthcare Cost and Utilization Project National Inpatient Sample (NIS) 2013 and OneFlorida 2013.

Figure 3 illustrates the prevalence of MCC stratified by sex. With respect to the number of MCCs, male and female older adults did not exhibit notable difference in both the OneFlorida and NIS data. No statistical tests were performed to test the statistical difference among the groups. This contrasted with the population aged 18 to 64 years in which women had a higher prevalence of MCCs.

### Prevalence of Multiple Chronic Conditions by Race or Ethnicity

**Figure 4:**
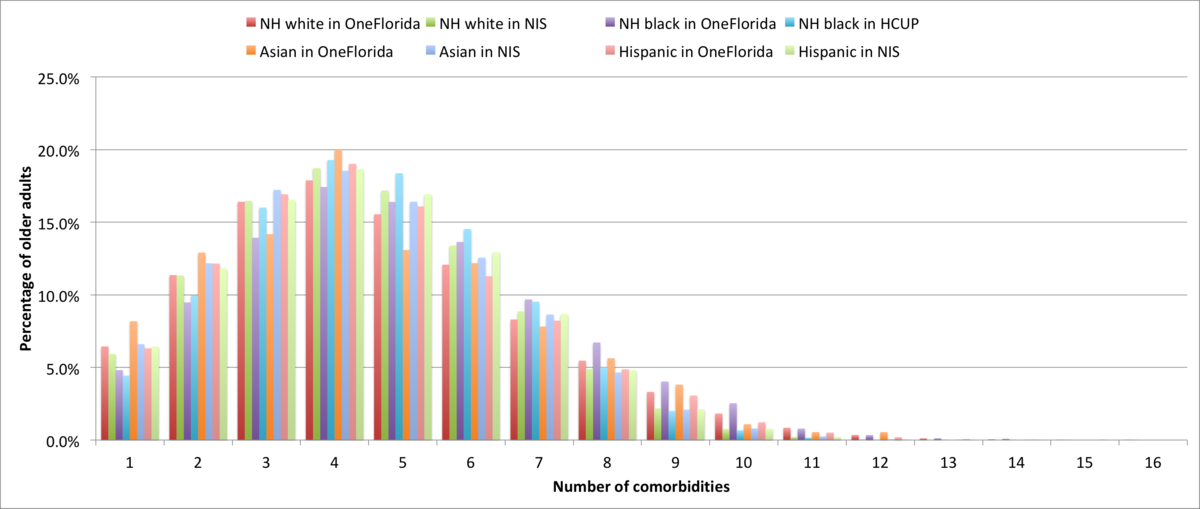
Prevalence of multiple chronic conditions in older adults by race and ethnicity groups in Healthcare Cost and Utilization Project National Inpatient Sample (NIS) 2013 and OneFlorida 2013 data. NH: non-Hispanic.

Figure 4 illustrates the prevalence of MCCs by race or ethnicity. It appears that the distribution of records with one or more CCs were similar among race or ethnicity groups. Note that even though Hispanic was overrepresented and Asian was underrepresented in OneFlorida, their MCC distribution within each race or ethnicity was similar to the NIS.

**Table 1:** Descriptive statistics for the National Inpatient Sample and OneFlorida patient datasets of older adults. HCUP NIS: Healthcare Cost and Utilization Project National Inpatient Sample.

Characteristics		OneFlorida 2013 (N=40,087)	OneFlorida 2012-2016 (N=147,900)	HCUP NIS 2013 (N=2,447,640)
Age in years, mean (SD)		76.4 (8.04)	76.4 (8.03)	78.0 (7.80)
**Gender, n (%)**				
	Male	19,094 (47.63)	71,642 (48.44)	1,084,593 (44.31)
	Female	20,993 (52.37)	76,255 (51.56)	1,362,844 (55.68)
	Unspecified	0 (0)	3 (0.0)	203 (0.01)
**Ethnicity, n (%)**				
	Non-Hispanic white	27,881 (69.55)	101,871 (68.88)	1,817,861 (74.27)
	Non-Hispanic black	5835 (14.56)	19,487 (13.18)	231,968 (9.48)
	Asian and Pacific Islander	550 (1.37)	1819 (1.23)	50,768 (2.07)
	Hispanic	3102 (7.74)	11,718 (7.92)	156,780 (6.41)
	Other	2719 (6.78)	13,005 (8.79)	190,263 (7.77)
Average number of multiple chronic conditions, (SD)		4.7 (2.3)	4.9 (2.6)	4.5 (2.0)

**Table 2:** Prevalence of the 25 chronic conditions in the 2013 National Inpatient Sample, the 2013 OneFlorida, and the pooled 2012 to 2016 OneFlorida data. HCUP NIS: Healthcare Cost and Utilization Project National Inpatient Sample.

Condition	Number of patients, n (%)		
	OneFlorida 2013 (N=40,087)	OneFlorida 2012-2016 (N=147,900)	HCUP NIS 2013 (N=2,447,640)
Hypertension	32,460 (80.97)	123,640 (83.60)	1,868,149 (76.32)
Hyperlipidemia	21,013 (52.42)	82,046 (55.48)	1,124,402 (45.94)
Ischemic heart disease	15,191 (37.90)	57,235 (38.69)	911,199 (37.23)
Diabetes	14,158 (35.32)	53,362 (36.07)	830,551 (33.93)
Anemia	14,445 (36.03)	57,108 (38.61)	819,538 (33.48)
Chronic kidney disease	12,525 (31.24)	49,957 (33.78)	813,196 (33.22)
Atrial fibrillation	9973 (24.88)	38,347 (25.93)	625,467 (25.55)
Heart failure	7926 (19.77)	31,411 (21.23)	620,787 (25.36)
Chronic obstructive pulmonary disease and bronchiectasis	8063 (20.11)	31,658 (21.41)	559,336 (22.85)
Rheumatoid arthritis or osteoarthritis	9325 (23.26)	41,348 (27.96)	481,299 (19.66)
Acquired hypothyroidism	7622 (19.01)	29,731 (20.10)	45,6024 (18.63)
Alzheimer disease and related disorders or senile dementia	5351 (13.35)	21,349 (14.43)	370,502 (15.14)
Depression	5643 (14.08)	22,554 (15.25)	323,717 (13.23)
Osteoporosis	2677 (6.68)	10,526 (7.12)	161,620 (6.60)
Asthma	3071 (7.66)	17,136 (11.59)	152,557 (6.23)
Stroke or transient ischemic attack	3040 (7.58)	13,288 (8.98)	117,165 (4.79)
Acute myocardial infarction	1799 (4.48)	7946 (5.37)	107,079 (4.37)
Prostate cancer	2516 (6.27)	10,146 (6.86)	103,151 (4.21)
Breast cancer	1876 (4.68)	7229 (4.89)	99,430 (4.06)
Alzheimer disease^a^	1202 (3.00)	4574 (3.09)	89,683 (3.66)
Colorectal cancer	1496 (3.73)	5398 (3.65)	77,409 (3.16)
Lung cancer	1376 (3.43)	5408 (3.66)	75,982 (3.10)
Hip or pelvic fracture	1127 (2.81)	5153 (3.48)	69,693 (2.85)
Benign prostatic hyperplasia	919 (2.39)	6297 (4.26)	47,979 (1.96)
Endometrial cancer	361 (0.90)	1419 (0.96)	15,173 (0.62)

^a^The case counts for persons with Alzheimer disease are also included in the counts for the Alzheimer disease and related disorders or senile dementia category.

**Table 3:** Prevalence of hypertension stratified by gender-age-racial or ethnic groups in the 2013 Healthcare Cost and Utilization Project (HCUP) National Inpatient Sample (NIS), the 2013 OneFlorida, and the pooled 2012 to 2016 OneFlorida data.

Sex	Age range in years	Race or ethnicity	Number of patients, n (%^a^)		
			OneFlorida 2013 (N=40,087)	OneFlorida 2012-2016 (N=147,900)	HCUP NIS 2013 (N=2,447,640)
Female	65-74	Asian and Pacific Islander	115 (0.29)	381 (0.26)	7487 (0.31)
Female	65-74	Hispanic	704 (1.76)	2696 (1.82)	28,310 (1.16)
Female	65-74	Non-Hispanic black	1516 (3.78)	5280 (3.57)	53,625 (2.19)
Female	65-74	Non-Hispanic white	4931 (12.30)	18,653 (12.61)	258,444 (10.56)
Female	75-84	Asian and Pacific Islander	84 (0.21)	308 (0.21)	8,888 (0.36)
Female	75-84	Hispanic	518 (1.29)	1949 (1.32)	27,484 (1.12)
Female	75-84	Non-Hispanic black	995 (2.48)	3464 (2.34)	42,002 (1.72)
Female	75-84	Non-Hispanic white	3871 (9.66)	14,522 (9.82)	275,781 (11.27)
Female	≥85	Asian and Pacific Islander	36 (0.09)	112 (0.08)	6295 (0.26)
Female	≥85	Hispanic	216 (0.54)	843 (0.57)	16,218 (0.66)
Female	≥85	Non-Hispanic black	574 (1.43)	1797 (1.22)	24,602 (1.01)
Female	≥85	Non-Hispanic white	2465 (6.15)	8765 (5.93)	226,926 (9.27)
Male	65-74	Asian and Pacific Islander	136 (0.34)	417 (0.28)	7355 (0.30)
Male	65-74	Hispanic	597 (1.49)	2428 (1.64)	25,393 (1.04)
Male	65-74	Non-Hispanic black	1240 (3.09)	4591 (3.10)	42,773 (1.75)
Male	65-74	Non-Hispanic white	5697 (14.21)	21,889 (14.80)	258,218 (10.55)
Male	75-84	Asian and Pacific Islander	59 (0.15)	245 (0.17)	6966 (0.28)
Male	75-84	Hispanic	417 (1.04)	1595 (1.08)	20,110 (0.82)
Male	75-84	Non-Hispanic black	710 (1.77)	2441 (1.65)	26,263 (1.07)
Male	75-84	Non-Hispanic white	3677 (9.17)	13,854 (9.37)	221,772 (9.06)
Male	≥85	Asian and Pacific Islander	20 (0.05)	78 (0.05)	3908 (0.16)
Male	≥85	Hispanic	133 (0.33)	560 (0.04)	8817 (0.36)
Male	≥85	Non-Hispanic black	234 (0.58)	815 (0.55)	9913 (0.41)
Male	≥85	Non-Hispanic white	1375 (3.43)	5449 (3.68)	120,645 (4.93)

^a^The denominator is all the patients ≥65 years old.

### Prevalence of Multiple Chronic Conditions by Pairs of Conditions

Table 4 shows the prevalence of the 10 most common pairs of co-occurring chronic conditions. Even though the prevalent MCCs of 2 conditions were the same in both OneFlorida and NIS cohorts, their rankings were slightly different. OneFlorida cohort had a higher percentage of patients with anemia and hypertension than the NIS cohort (32.17% vs 25.79%). OneFlorida cohort had a slightly higher percentage of older adults with atrial fibrillation and hypertension than the NIS cohort (22.72% vs 19.88%). The most prevalent MCCs of 2 conditions among the 3 datasets were—hyperlipidemia and hypertension, hypertension and ischemic heart disease, diabetes and hypertension, chronic kidney disease and hypertension, anemia and hypertension, and hyperlipidemia and ischemic heart disease.

### Trends in Multiple Chronic Conditions

**Figure 5:**
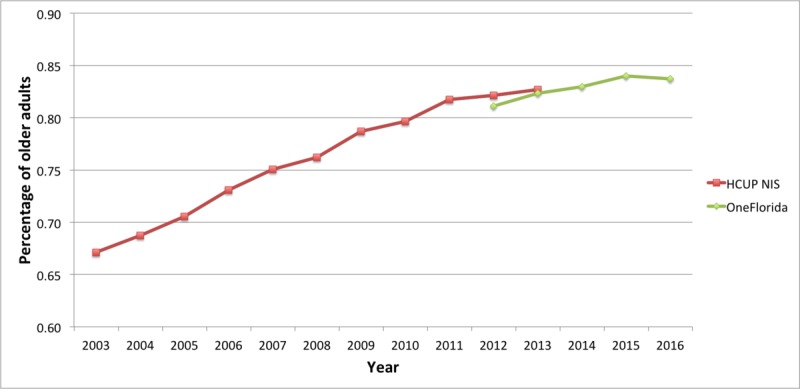
Trends of multiple chronic conditions in older adults for the Healthcare Cost and Utilization Project (HCUP) National Inpatient Sample (NIS; 2003-2013) and OneFlorida (2012-2016). The denominator in the prevalence is the total number of older adults with at least one of the 25 conditions in each year.

**Figure 6:**
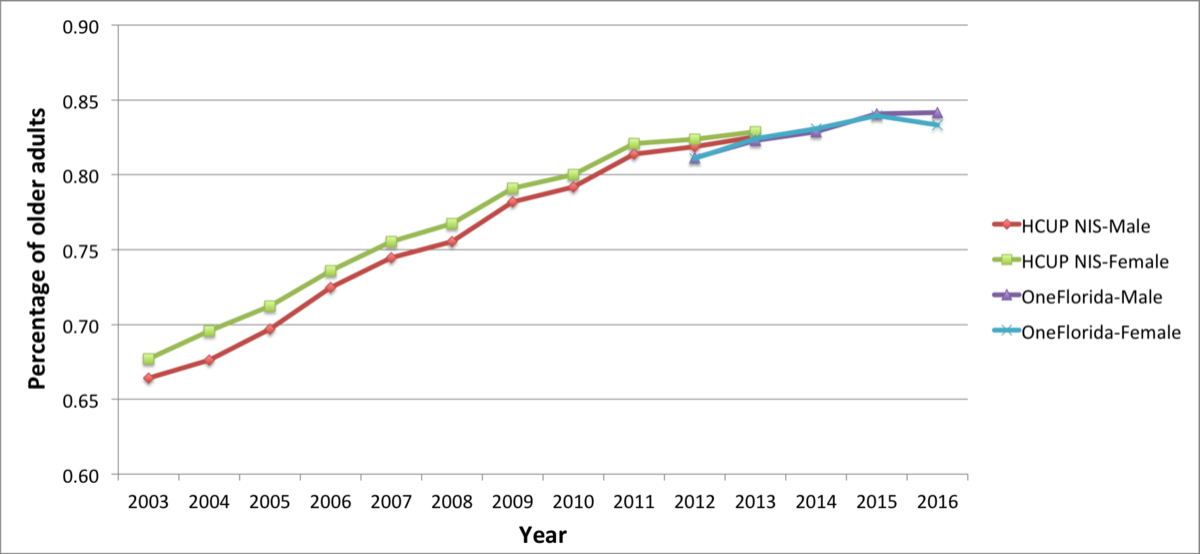
Trends of multiple chronic conditions in older adults by gender for the Healthcare Cost and Utilization Project (HCUP) National Inpatient Sample (NIS) (2003-2013) and OneFlorida (2012-2016). The denominator in the prevalence is the total number of older male or female with at least one of the 25 conditions in each year.

**Figure 7:**
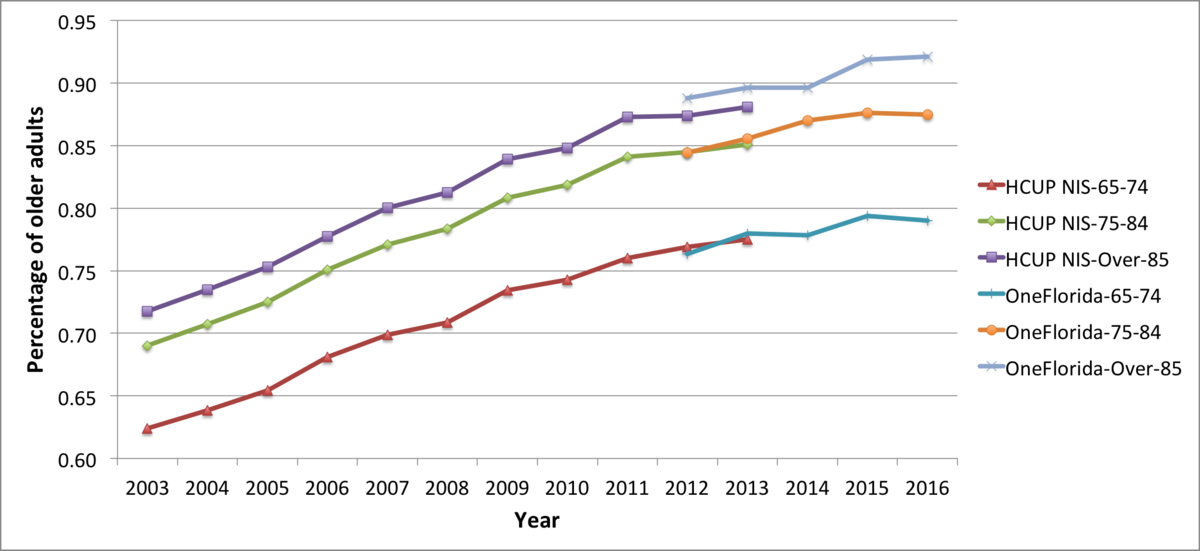
Trends of multiple chronic conditions in older adults by age group for the Healthcare Cost and Utilization Project (HCUP) National Inpatient Sample (NIS) (2003-2013) and OneFlorida (2012-2016). The denominator in the prevalence is the total number of older adults in each age group with at least one of the 25 conditions in each year.

**Figure 8:**
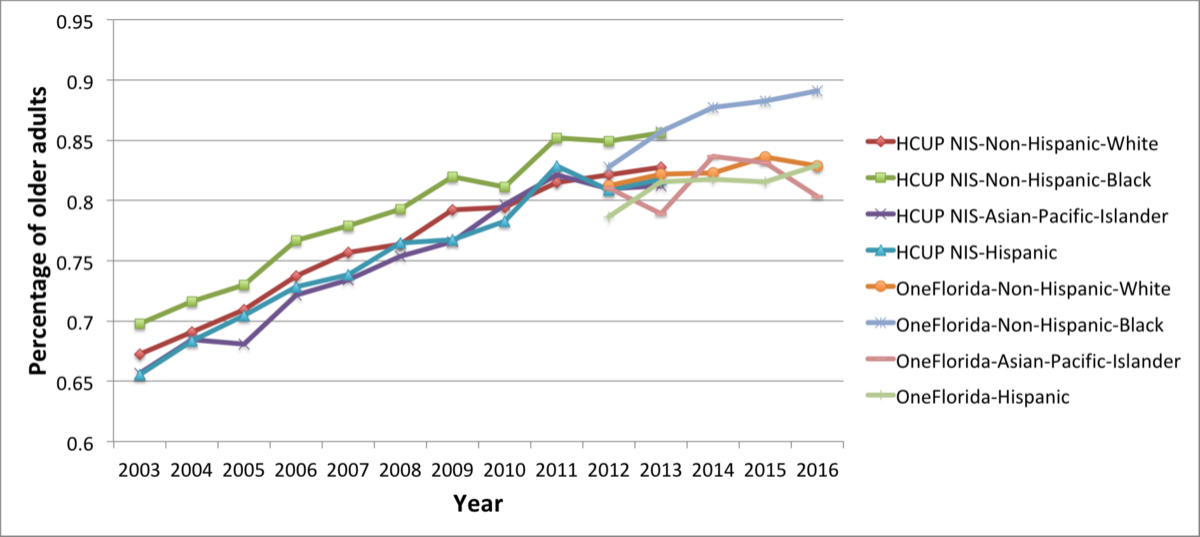
Trends of multiple chronic conditions in older adults by racial-ethnic groups for the Healthcare Cost and Utilization Project (HCUP) National Inpatient Sample (NIS; 2003-2013) and OneFlorida (2012-2016). The denominator in the prevalence is the total number of older adults in each racial-ethnic group with at least one of the 25 conditions in each year.

The following 4 figures (Figures 5-8) present a longitudinal examination of the number of discharges reflecting 2 or more conditions for the period 2012 to 2016 for the OneFlorida data and 2003 to 2013 for the NIS data. In Figure 5, the overall prevalence of 2 or more CCs raised steadily from approximately 66% in 2003 to approximately 83% in 2013 in the NIS data. The OneFlorida data began in 2012 at approximately 81% prevalence of MCC and rose to approximately 84% by 2016. Both slopes showed a monotonic increasing trend in the prevalence of MCCs.

The slope of the MCC prevalence by gender in Figure 6 appeared to be very similar to the overall slope in Figure 5. The slopes for males and females in the NIS data were parallel with 1% to 2% difference for males and females and ultimately converged at approximately 84% by 2013. Pearson correlation coefficient showed a strong positive correlation between male and female older adults with an *R* value of .9966. The lines for OneFlorida data for males and females were nearly coincident and appeared to continue the slope of the NIS data.

In Figure 7, the prevalence of MCC by age group is presented for NIS and OneFlorida data. The NIS slopes for the 3 age groups were parallel through 2013. Pearson correlation coefficient showed a strong positive correlation among the 3 age groups—the *R* value between NIS 65 to 74 years age group and 75 to 84 years age group was .9972; the *R* value between NIS 65 to 74 years age group and NIS over 85 years age group was .9961. Nevertheless, there was about an 8-percentage point difference between the youngest age group (65-74 years) and the middle age group (75-84 years). The oldest age group (over 85 years) appeared to be about 4 percentage points higher than the 75 to 84 years age group throughout the time range. Similar differences were seen between the parallel slopes for OneFlorida data, although the 85 years and over group was trending somewhat higher as compared with the same age group in the NIS.

Finally, in Figure 8, we present the prevalence of MCCs by racial-ethnic groups. The general trend was the same as seen in Figures 5-7. The non-Hispanic black and non-Hispanic white groups ran parallel with the black group averaging about 2 percentage points higher. The Pearson correlation coefficient showed a strong positive correlation between non-Hispanic black and non-Hispanic white groups with an *R* value of .9959. The Hispanic group and the Asian and Pacific Islander group, both averaged a bit lower than the non-Hispanic white population, but there was more volatility probably due to smaller sample size. This was particularly true for the OneFlorida data.

**Table 4:** Prevalence of the 10 most common pairs of co-occurring chronic conditions. HCUP NIS: Healthcare Cost and Utilization Project National Inpatient Sample.

Condition A	Condition B	Number of patients, n (%)		
		OneFlorida 2013 (N=40,087)	OneFlorida 2012-2016 (N=147,900)	HCUP NIS 2013 (N=2,447,640)
Hyperlipidemia	Hypertension	19,452 (48.52)	73,732 (49.85)	960,388 (39.23)
Hypertension	Ischemic heart disease	16,672 (41.58)	56,945 (38.50)	745,865 (30.47)
Diabetes	Hypertension	13,410 (33.45)	49,079 (33.18)	698,256 (28.53)
Chronic kidney disease	Hypertension	11,727 (29.25)	45,482 (30.75)	671,397 (27.43)
Anemia	Hypertension	12,898 (32.17)	49,566 (33.51)	631,247 (25.79)
Hyperlipidemia	Ischemic heart disease	12,041 (30.04)	43,100 (29.14)	530,768 (21.68)
Heart failure	Hypertension	7452 (18.59)	28,625 (19.35)	490,243 (20.03)
Atrial fibrillation	Hypertension	9109 (22.72)	33,851 (22.89)	486,609 (19.88)
Diabetes	Hyperlipidemia	10,738 (26.79)	38,023 (25.71)	449,597 (18.37)
Hypertension	Chronic obstructive pulmonary disease and bronchiectasis	7180 (17.91)	27,285 (18.45)	414,983 (16.95)

## Discussion

### Principal Findings

The main objective of our study was to compare the prevalence of common CCs and MCCs in older adults in the Florida and US national population using the OneFlorida Data Trust and the NIS of HCUP. The results showed that CCs and MCCs were prevalent in older adults, both nationally and in the Florida population. The most prevalent CCs were the same for older adults in the OneFlorida Data Trust and HCUP NIS. For hypertension, the largest differences in the estimates between the 2013 OneFlorida Data Trust and NIS were about merely 3% for non-Hispanic white females 85 years and older and males 65 to 74 years old. Regarding the number of MCCs, OneFlorida Data Trust and NIS did not exhibit any notable difference with respect to gender and race or ethnicity. The most prevalent MCCs of 2 CCs were also the same for OneFlorida 2013, NIS 2013, and OneFlorida 2012 to 2016. With regard to the MCC trends, the slopes of the increasing trend in the number of discharges reflecting 2 or more conditions appeared quite similar in both data sources. With respect to age group, the oldest age group (over 85 years of age) appeared to be about 4 percentage points higher than the 75 to 84 years age group and 12 percentage points higher than the 65 to 75 years age group throughout the time range. Even though slight differences were observed, similar estimates of prevalence of CCs and MCCs across OneFlorida Data Trust and NIS showed that large clinical research networks such as OneFlorida provide rich data resources for conducting large-scale secondary data analyses.

Although the MCC prevalence presented in this study is generalizable to the older US adults in the noninstitutionalized national population, the use of OneFlorida Data Trust and the HCUP NIS has limitations. OneFlorida and NIS both only captured the conditions that were confirmed by a doctor or health professionals in inpatient settings, potentially leading to the underrepresentation of conditions that remain undiagnosed or were not recorded in the inpatient care (eg, arthritis [3]). Many uninsured adults would not get into these databases until 65 years of age when they become eligible for Medicare. Undocumented immigrants would never make it into Medicare. For example, the prevalence of arthritis reported in a self-reported national survey almost doubles the prevalence of arthritis in the inpatient clinical data reported in Table 2. Of the conditions captured, we only used the CCW algorithm from the Centers for Medicare and Medicaid Services (CMS) and considered a single occurrence of the diagnosis code of a particular condition when identifying patients who had such a condition. There might be false positive cases included in the analysis. Furthermore, although the OneFlorida Clinical Research Consortium [41] covers care for approximately 48% Floridians, the consortium is missing representations from a few of the key health care markets in Florida, such as Tampa, and cities in the Florida panhandle area. Moreover, the prevalence of CCs might be overestimated for Florida, as there might be duplicated patient records across the different health care organizations in the OneFlorida consortium. For example, EHRs from health care providers and claims data from payers can have records for the same patient. In addition, the same patient can seek care in different health care organizations in the network. Thus, linking related data and resolving duplicates in a clinical research network is a significant task in improving the quality of a dataset. In our recent effort, we have linked and deduplicated patient records across 2 of the data sources in the OneFlorida consortium—University of Florida Health system and Florida Medicaid. We eliminated 430,106 duplicate patient records across these 2 sources, which is approximately 6.4% of the Florida Medicaid population.

Our study confirmed the previous literature [5] and showed the increasing trend in the prevalence of MCCs among the older US adults. We also showed that the characteristics of the patient population in these clinical research networks such as OneFlorida are comparable to national-level sample data. Furthermore, these clinical research networks have integrated fine-grained details of the patients (eg, encounters, procedures, diagnoses, medications, lab results, as well as patient-reported outcomes) from multiple health care organizations, which can provide a more complete picture of the patients’ health traits. Enabled by clinical research networks such as OneFlorida, large-scale secondary data analyses can be conducted to discover novel findings in biomedical research, such as sophisticated relationships among diseases, medications, vital signs, adverse events, and outcomes.

### Implication and Future Directions

The OneFlorida Data Trust is the informatics infrastructure that supports pragmatic trials, comparative effectiveness research, implementation science, and other research in the OneFlorida Clinical Research Consortium. The most key research functions supported by OneFlorida and PCORnet include cohort discovery and participant enrollment, recognizing the barriers in identifying and recruiting research participants for clinical research studies, especially for rare diseases. Furthermore, the population representativeness of clinical research has long been a concern [42]. Particularly, older adults are widely reported to be underrepresented in clinical studies across major medical conditions such as cardiovascular diseases [43,44], cancer [45,46], dementia [47], and diabetes [48,49]. Due to the lack of evidence in the clinical practice guideline in treating older adults with MCCs, it is imperative to generate such evidence by involving older adults with normal age-related organ impairment and comorbid conditions that may not interact with the treatment under study. However, older adults are often unfairly excluded by restrictive eligibility criteria in clinical studies [46,50]. Meanwhile, MCCs are most prevalent in the Medicare population. Persons with MCCs are at an increased risk of mortality, morbidity, hospitalization, high medical costs, and adverse events [51]. In order to understand how older adults with MCCs are represented in clinical trials, it is important to understand the prevalence of MCCs in older adults. In future work, we will use laboratory test results and medications to enhance the sensitivity and specificity of case assignment for some conditions. We will also compare the outpatient data of OneFlorida Data Trust with the national outpatient databases such as the Nationwide Emergency Department Sample.

## Abbreviations

CC: chronic condition
CCW: chronic condition warehouse
CDM: common data model
CDRN: Clinical Data Research Network
CMS: Center for Medicare and Medicaid Services
EHR: electronic health record
HCUP: Healthcare Cost and Utilization Project
HITECH: Health Information Technology for Economic and Clinical Health
ICD: International Classification of Diseases
MCC: multiple chronic conditions
NH: non-Hispanic
NIS: National Inpatient Sample
PCORnet: Patient-Centered Clinical Research Network
PCORI: Patient-Centered Outcomes Research Institute
RQ: research question
